# Condition-associated modulation of melanin-based skin pigmentation in juvenile Atlantic salmon (*Salmo salar*)

**DOI:** 10.1098/rsos.260816

**Published:** 2026-10-28

**Authors:** Mikkel Gunnes, Evelina A. L. Green, Inger Lise Andersen, Øyvind Øverli

**Affiliations:** Department of Animal and Aquacultural Sciences, Faculty of Biosciences, Norwegian University of Life Sciences, Ås, Akershus, Norway

**Keywords:** Atlantic salmon, melanin-based pigmentation, phenotypic plasticity, condition-dependent traits, stress responsiveness, coping style, feeding behaviour, adaptation to novelty

## Abstract

Melanin-based pigmentation in vertebrates has often been regarded as a stable trait, inexpensive to produce and highly heritable. Accumulating evidence, however, links melanin patterns to behavioural, physiological and endocrine variation. In salmonids, eumelanin-based skin spottiness has been associated with stress responsiveness and coping style, but it remains unclear to what extent environmental challenge induces within-individual state-dependent changes in spot expression. Here, we examined whether feeding dynamics during acclimation to novelty are associated with longitudinal changes in visible skin spot density in juvenile Atlantic salmon (*Salmo salar*), in which dark patterns are expressed through eumelanin-containing melanophores. Fish were transferred from group rearing to individual housing, inducing transient feeding suppression followed by large inter-individual variation in feeding recovery and growth. Although visible spot density declined overall, the magnitude and direction of change varied among individuals and covaried with feed intake, growth and condition factor. Individuals with higher feed intake and growth, and smaller declines in condition factor, exhibited reduced spot loss and, in some cases, increased spot density, whereas lower-performing individuals showed larger reductions. Pre-existing spot patterns predicted post-transfer performance. These findings suggest that salmonid skin spots track resource acquisition and coping capacity, consistent with regulated phenotypic plasticity in condition-associated visible pigmentation.

## 1. Introduction

Animal pigmentation patterns have inspired numerous influential topics in evolutionary biology, including sexual selection, aposematism, crypsis, mimicry and signalling [1–7]. Pigmentation patterns have also been demonstrated to play a pivotal role in sympatric speciation, driving diversification of populations through assortative mating and ecological differentiation [1,8–10].

Vertebrate coloration arises primarily from two pigment groups: carotenoids and melanins [11–13]. Carotenoids, conferring yellow, orange and red hues, cannot be synthesized de novo by vertebrates and must be acquired through dietary sources [14–17]. Thus, their expression often mirrors nutritional status and resource acquisition, supporting their proposed role as condition-dependent signals of individual quality under selection [18–20]. The physiological roles of carotenoids, including anti-oxidant defence, immune modulation and homoeostatic support, reinforce their use as honest signals in mate choice [21–25].

Unlike carotenoid-based coloration, melanin-based pigmentation has often been considered more strongly influenced by endogenous genetic regulation of pigment biosynthesis and deposition [9,26,27]. Melanins occur primarily as eumelanin, which produces black to brown pigmentation, and pheomelanin, which produces reddish to yellowish pigmentation [28,29]. Consistent with this genetic framing, melanin-based traits have frequently been described as relatively heritable, metabolically inexpensive to synthesize and stable across environmental conditions [5,30–35]. This has contributed to the view that melanin-based patterns may be weaker indicators of an individual's ability to obtain resources and cope with their environment (i.e. ‘quality’) compared to carotenoid pigmentation. However, this distinction is not absolute. Melanin-based coloration has also been repeatedly associated with a diverse array of phenotypically plastic traits and processes, including behaviour, physiology, endocrine regulation and life-history characteristics across animal taxa [36–41]. Accordingly, melanin-based traits are increasingly understood as emerging from interactions between genetic architecture, environmental conditions and physiological regulation. These interactions may include developmental and nutritional influences, as well as epigenetic and epitranscriptomic mechanisms through which pigment expression can be modulated across vertebrate taxa [42–48].

In land-living vertebrates, melanin-based traits are not only linked to indicators of health, vigour and fitness but are also correlated with key physiological and endocrine factors such as immune responsiveness, body mass and stress coping ability [49–57]. Examples include associations with personality, stress responsiveness, and immunity in birds [49,58–61] and resource-holding potential and immune response in reptiles [62,63]. Additional studies support the view that melanin-based traits are associated with underlying physiological strategies for coping with energetic constraints, parasitism and environmental stress [64,65].

Strong melanin patterning may also reflect social dominance, aggression and other personality traits associated with distinct coping styles [36]. These associations are consistent with both pleiotropic effects and functional integration between pigmentation loci and physiological or endocrine pathways [36,41,66,67]. In species where melanin is not primarily used for camouflage, contrast-rich markings often serve as visual signals in social and reproductive contexts [59,60,68–72]. Classic cases include the breast stripe of male great tits (*Parus major*) as a badge of social status [59] and the dark mane of lions (*Panthera leo*) as a cue to male health, status and mating potential [72]. Against this background, external melanin-based pigmentation has been proposed as a morphological trait conveying information about individual state [40,73]. This idea is motivated by long-standing interest in externally visible traits that reflect stress responsiveness and condition in animals [36,39,41]. However, while previous work has established that melanin-based traits are associated with environmental conditions and physiological performance [40,41,74], much of this work has primarily focused on differences among individuals, genotypes or colour morphs. Although dietary factors can influence the capacity for melanin synthesis [75], less is known about whether visible melanin-based pigmentation changes dynamically within individuals over time in response to variation in resource acquisition and performance.

Owing to their high degree of developmental and phenotypic plasticity [76,77], teleost fishes provide a particularly useful model for studying variation in skin pigmentation patterns and their association with physiological and behavioural flexibility. Teleost melanophores synthesize eumelanin, whereas pheomelanin appears not to be produced in fish melanophores. Accordingly, black, grey and dark brown pigmentation in teleost skin is generally attributed to eumelanin-containing melanophores [78,79]. In salmonids, dark skin spots are generally interpreted as eumelanin-based melanophore patterns [80]. Unlike feathers and hair, in which melanin becomes embedded in keratinized structures during growth and is largely fixed until replacement, fish pigmentation is expressed through living chromatophores in the skin, scales and fins. Melanophores can alter visible pigmentation through redistribution of melanin-containing organelles (melanosomes) within the cells, enabling physiological colour change over shorter timescales, from seconds to minutes, whereas longer-term changes in melanophore number or distribution occur over days to weeks [81–84].

As in other vertebrate groups, persistent variation in melanin-based skin pigmentation in fishes has been linked to social dominance, aggression, boldness, stress responsiveness, latency to resume normal behaviour after stress and increased resistance to infections [85–90]. Further, melanic individuals are often preferred in mating [91,92]. Artificial selection on glucocorticoid (cortisol) responsiveness in salmonids has induced microevolutionary changes in melanin spot expression, tying endocrine mechanisms to pigmentation. Individuals with low stress responsiveness exhibit consistently higher spot density than high responders [88]. Moreover, melanic coloration has been associated with reproductive success and offspring viability [93,94]. Similarly, in farmed rainbow trout (*Oncorhynchus mykiss*), melanin-based skin spots are moderately heritable and show a positive genetic correlation with growth, suggesting that selection for body weight can indirectly enhance spottiness [95].

Environmental factors can also influence melanin-based pigmentation in fishes. In teleost fishes, including salmonids, changes in substrate or tank colour induce reversible adjustments in overall skin coloration [80]. In Atlantic salmon, for instance, rearing conditions can lead to striking (six- to sevenfold) differences in spot patterns, surpassing the effects of domestication or genetic background [96]. The extent to which other environmental factors contribute to modulating the genetic programming of pigment expression in fishes remains unknown.

In summary, in the salmonid model system, melanin-based skin spottiness has been associated with variation in stress responsiveness and coping style, yet it remains unresolved whether these pigmentation patterns primarily reflect fixed variation or act as a dynamic indicator of individual condition. Transfer to a novel environment commonly induces transient suppression of feeding in salmonids, followed by pronounced individual differences in latency and extent of feeding recovery (e.g. [97–99]). Such variation is closely linked to stress coping style, with proactive individuals typically resuming feeding more rapidly and maintaining higher feeding motivation under challenge than reactive individuals [100–103]. Because coping style has also been linked to melanin-based pigmentation in salmonids [88], the question arises as to whether feeding performance following exposure to environmental novelty is associated with within-individual changes in visible melanin-based spot density.

In the present study, we investigated melanin-based skin spot density following transfer from group rearing to individual housing in juvenile Atlantic salmon. We tested whether both baseline spot density and within-individual changes in visible spot density were associated with individual differences in feeding recovery and performance. Specifically, we predicted that individuals with higher initial spot density would exhibit higher feed intake and growth following transfer, and that individuals with higher feed intake, growth and condition factor would show greater retention or increases in visible skin spot density over time during acclimation to individual housing.

## 2. Material and methods

### 2.1. Animal husbandry

Fertilized eggs of Atlantic salmon (*Salmo salar*) were obtained from a commercial breeder (AquaGen AS, Trondheim, Norway), hatched and reared at the experimental fish facility. Fish were maintained for approximately 1 year prior to the experiment. During the six months preceding the study, fish were maintained in three separate circular 340 l tanks connected to a recirculating aquaculture system (RAS). Stocking density was adjusted over time through sorting and redistribution of individuals to maintain appropriate rearing conditions as the fish grew. At the time of experimental initiation, a total of 120 fish were held in these tanks (*n* = 40 per tank), corresponding to a rearing density of 2.3 kg m^−3^. Water temperature ranged from 12.8 to 14.7°C (seasonally variable), oxygen saturation remained consistently above 85%, with a total system flow of 110–135 l min^–1^ and water exchange rates of 4–7 l min^–1^. Fish were fed daily using standard facility feeding protocols corresponding to an expected growth rate of approximately 1% per day, using 3 mm commercial pellets (Nutra Olympic, Skretting AS, Stavanger, Norway). Fish were maintained under a 24 h light regime. From this population, 12 juvenile fish within a size range suitable for individual housing (weighing 229.7 ± 49.43 g, mean ± s.d.) were netted from the holding tanks and transferred to individual compartments in glass aquaria for observation. Selection was not based on pigmentation, feeding behaviour, sex or expected performance.

### 2.2. Experimental set-up

The experiment was conducted in a controlled laboratory setting with glass aquaria (100 × 50 × 50 cm), supplied by the same RAS as the holding tanks. Each aquarium was divided into four 50 l compartments (25 × 50 × 50 cm) using opaque PVC partitions to prevent physical and visual contact between fish. One fish was randomly assigned to each compartment.

The fish were netted from the holding tanks and gently anesthetized in 50 mg l^–1^ AQUI-S (AQUI-S®, Lower Hutt, New Zealand) for measurements of body mass (± 1 g) and fork length (± 1 mm). Photographs of both sides of each fish were captured using the integrated wide-angle camera of an iPhone 12 (Apple Inc., Cupertino, CA, USA) under ambient indoor lighting for subsequent analysis of baseline melanin-based skin spot density. The camera system features a 12-megapixel CMOS sensor (pixel size 1.4 µm) with a fixed f/1.6 aperture and a 26 mm equivalent focal length. Total handling time per fish, including anaesthesia and recovery, was approximately 5 min, during which fish were held out of water for approximately 1 min during measurements and imaging. Fish were then transferred to the individual compartments and allowed to acclimate for 21 days.

Water temperature during the experiment ranged from 14.0 to 16.6°C, and oxygen saturation remained above 85% consistently. Continuous 24 h illumination was provided by LED strips mounted approximately 50 cm above the water surface. The holding tanks used prior to the experiment had white interiors, whereas the experimental aquaria had a uniform medium-grey background. Changes in background coloration are known to induce adjustments in overall skin pigmentation in teleost fishes [80].

Beginning 24 h after transfer, fish were hand-fed once daily between 09.00 and 13.00 using the same pellets as in the holding tanks. Pellets were offered individually until refusal of three consecutive pellets (satiety) or until a maximum of 1% of individual body mass per day, calculated from body mass measured at the start of the experiment, was reached. Food intake was recorded daily. After 14 days, feeding frequency was reduced to every second day to minimize disturbance. Fish were monitored daily for health and behaviour, and no mortality occurred during the experiment.

### 2.3. Endpoint measurements

At the conclusion of the experimental period, fish were netted from their individual compartments and euthanized by immersion in a lethal dose of tricaine methane-sulfonate (800 mg l^−1^ MS-222; Finquel® Vet, MSD Animal Health Norge AS) buffered to pH approximately 7.0 with sodium bicarbonate (NaHCO_3_). Immediately following euthanasia, body mass and length were measured, and each fish was photographed for assessment of endpoint melanin-based skin spot density and potential spot density changes (see §2.4 for details). Sex was determined by visual inspection of the gonads (*n* = 7 males, *n* = 5 females).

### 2.4. Image analysis

Preliminary inspection revealed substantial inter-individual variation in the number, size and contrast of melanin spots on the operculum. By contrast, individual spots on the dorsal region of the body were more consistent in size, appearance and contrast across individuals and time points, while spot number within this region varied among individuals. Accordingly, pigmentation analysis focused on a standardized dorsal region of interest (ROI) between the operculum and the base of the dorsal fin, consistent with previous studies that quantified melanin-based skin spots on the body of salmonids [88,104]. The terms melanin spot density, melanin spottiness and melanin spot expression refer specifically to the number of visually detectable spots within the defined ROI and do not represent total melanin content, melanophore number or pigment concentration. Spot counts were based on visually detectable contrast between melanin spots and surrounding skin, as assessed by the human observer under standardized viewing conditions.

### 2.5. Image preprocessing

To standardize contrast and reduce variation in lighting and image intensity across samples, all images were pre-processed using contrast limited adaptive histogram equalization (CLAHE) in ImageJ (v.2.16.0; [105]). A block size of 63 pixels, 256 histogram bins and a clip limit of 2 were applied uniformly. Preprocessing was performed prior to image scaling, ROI delineation and manual quantification of melanin spots.

Individuals were analysed in a randomized order by a single observer. For each individual, left- and right-side images at baseline and endpoint were paired, and time points were quantified sequentially to maintain within-individual consistency. Blinding was not possible owing to visible morphological features and background-dependent changes in pigmentation.

### 2.6. Scaling and region of interest measurements

Baseline and endpoint images of the same side of the same fish were opened simultaneously in ImageJ, and the image scale was converted from pixels to cm using a known distance on the scale bar and applying the Set Scale function. The image showing the smallest visible dorsal extent was used to define the ROI to ensure consistent coverage across both time points. A polygonal ROI was drawn manually using anatomical landmarks, with horizontal bounds from the posterior margin of the operculum to the anterior base of the dorsal fin and vertical bounds from the lateral line to the dorsal margin of the body (figure 1). Reference landmarks along the ROI edges were used to guide placement of an identical ROI in the corresponding image (baseline/endpoint) to ensure spatial consistency. ROI area (cm^2^) was recorded using Analyze > Measure in ImageJ, and minor adjustments constrained to anatomical landmarks were made where necessary to align ROIs across paired images. Images were saved with the ROI stored as an overlay for subsequent spot quantification.

**Figure 1.**
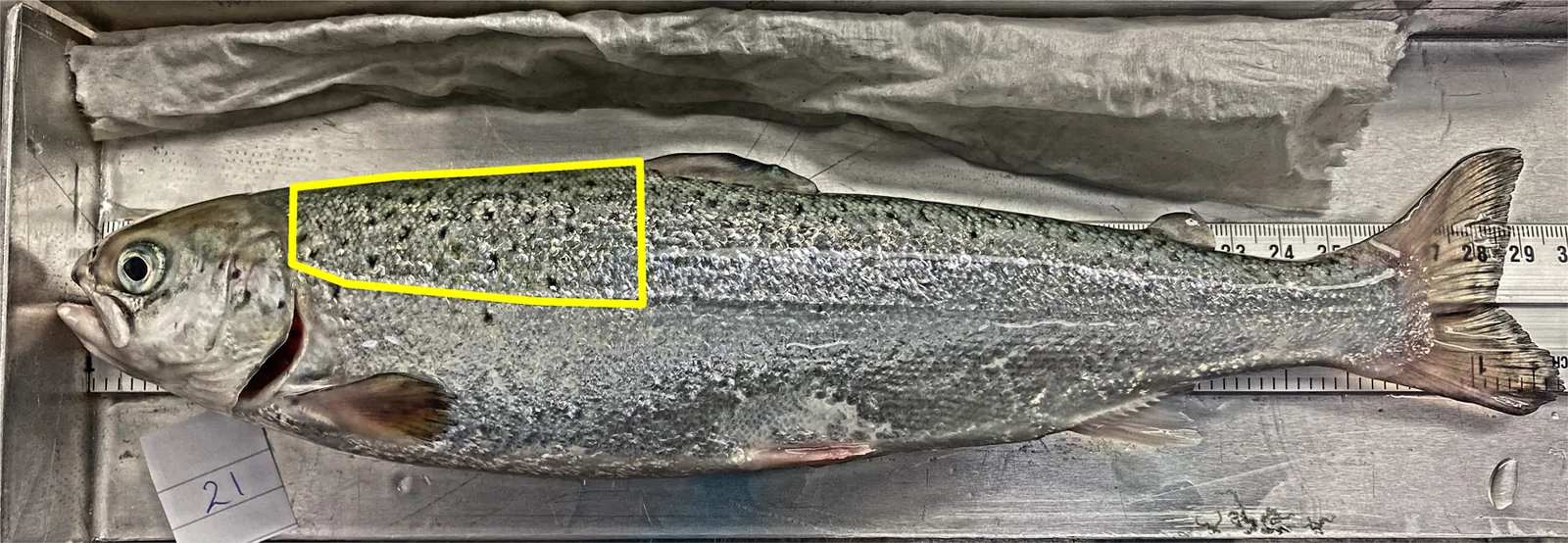
Location of the standardized region of interest (ROI) used for quantification of melanin-based skin spot density. The yellow polygon indicates the dorsal body region between the posterior margin of the operculum and the anterior base of the dorsal fin used for spot counting. This region was selected owing to its relatively consistent spot size and contrast compared to other body areas and follows approaches used in previous studies [88,104].

### 2.7. Spot counting and output files

The number of visible melanin spots within each ROI was manually counted using the Cell Counter plugin in ImageJ (RRID:SCR_025376) and converted to spot density by dividing by the ROI area. The ROI outline was saved as an overlay, and images with annotated spot markers were exported for visualization. All raw images, ROI overlays and annotated images were retained for verification and downstream analysis.

### 2.8. Statistical analysis

All analyses were conducted in R Studio (v.4.5.3; R Core Team, Vienna, Austria) using the packages ‘ggplot2’ for visualization, ‘dplyr’ and ‘tidyr’ for data handling, ‘patchwork’ for figure assembly, ‘broom’ for model output extraction and ‘svglite’ for figure export.

To describe feeding dynamics following transfer to isolation, daily feed intake was summarized across individuals over the observation period. Changes in body weight, condition factor (Fulton's *K*) and skin spot density between the start and end of the experiment were assessed using paired tests. Condition factor (*K*) was calculated as *K* = 100 × body mass (g) / fork length^3^ (cm). Normality of paired differences was evaluated using the Shapiro–Wilk test. Paired *t*-tests were applied to normally distributed variables (*K* and spot density), while body weight was analysed using a Wilcoxon signed-rank test. Central tendencies and dispersion are presented graphically, with baseline values summarized in the text.

Associations between post-transfer performance and pigmentation change were assessed using linear regression, with relative change in skin spot density (% of baseline) as the response variable. Separate models were fitted for growth (% change in body weight), accumulated feed intake (% of initial body weight) and change in condition factor (% of initial *K*). Model assumptions (linearity and homoscedasticity) were evaluated visually using residual-versus-fitted and quantile–quantile plots.

To test whether baseline pigmentation predicted subsequent performance, simple linear regression models were fitted with initial skin spot density (spots cm^−2^) as the predictor and growth, cumulative feed intake and change in condition factor as response variables. Additional models assessed associations between baseline spot density and initial body weight and condition factor.

The potential influence of initial body weight was evaluated using linear regression models relating initial body weight to performance metrics and pigmentation change. Multiple linear regression models were then fitted to test whether associations between performance metrics and pigmentation change persisted after accounting for initial body weight. Separate models were constructed for each performance metric.

Sex differences in pigmentation change and performance metrics were assessed using Wilcoxon rank-sum tests.

For regression models, effect sizes are reported as unstandardized coefficients (*b*) with 95% confidence intervals (CI). Model fit is reported as *R*^2^ or adjusted *R*^2^ as appropriate. All tests were two-tailed with a significance level of *α* = 0.05, and raw *p*-values are reported without formal corrections for multiple comparisons.

## 3. Results

### 3.1. Feeding dynamics, growth and changes in pigmentation

At the start of the observation period, condition factor ranged from 0.99 to 1.27 (1.16 ± 0.07; mean ± s.d.), and spot density ranged from 3.02 to 5.88 spots cm^−2^ (4.44 ± 0.89). All individuals ceased feeding immediately after transfer to individual housing, and latency to resume feeding ranged from 3 days to the entire observation period (figure 2). After commencing feeding, daily feed intake increased gradually, with marked inter-individual variation in feeding trajectories and cumulative feed intake over the observation period (range: 0–16% initial body weight; figure 2). Individuals that failed to resume feeding exhibited minimal or negative growth, whereas higher-performing individuals drove the overall increase in body mass.

**Figure 2.**
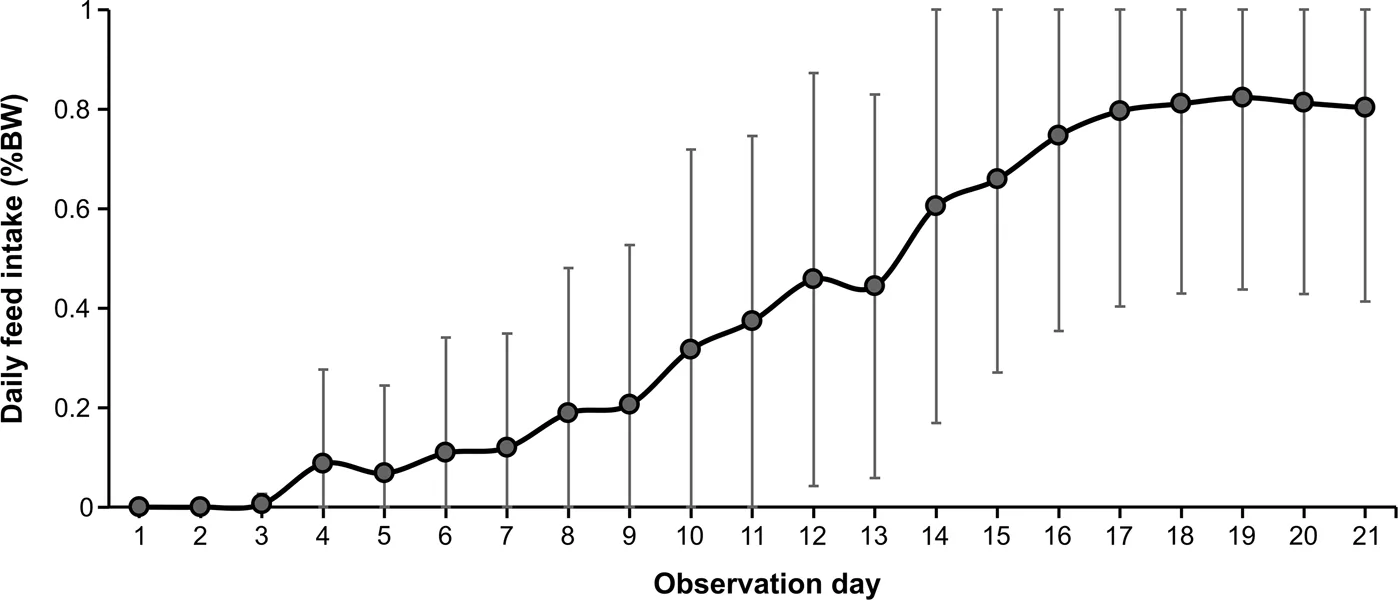
Development of feed intake in *n* = 12 juvenile Atlantic salmon after transfer to individual housing. Curve shows average daily feed intake (as a percentage of initial body weight d^−1^) over a 21-day observation period following transfer from group rearing to individual compartments in aquaria. Points represent daily averages across individuals, and error bars indicate ± 1 s.d., illustrating inter-individual variation in latency to feeding resumption and subsequent feeding trajectory. Error bars are truncated at the lower and upper limits of possible daily feed intake values shown in the figure, 0 and 1% body weight d^−1^, respectively.

Despite the initial appetite suppression, mean body mass increased significantly over the observation period (*V* = 67, *p* = 0.027; figure 3A). By contrast, condition factor (*t*_11_ = −4.12, *p* = 0.002; figure 3B) and spot density (*t*_11_ = −2.72, *p* = 0.02; figure 3C) decreased between the start and end of the study. Fish exhibited a visible darkening of overall skin coloration following transfer to the grey-background aquaria, consistent with background adaptation in teleosts. Despite this, visible melanin-based skin spot density declined in most individuals over the observation period.

**Figure 3.**
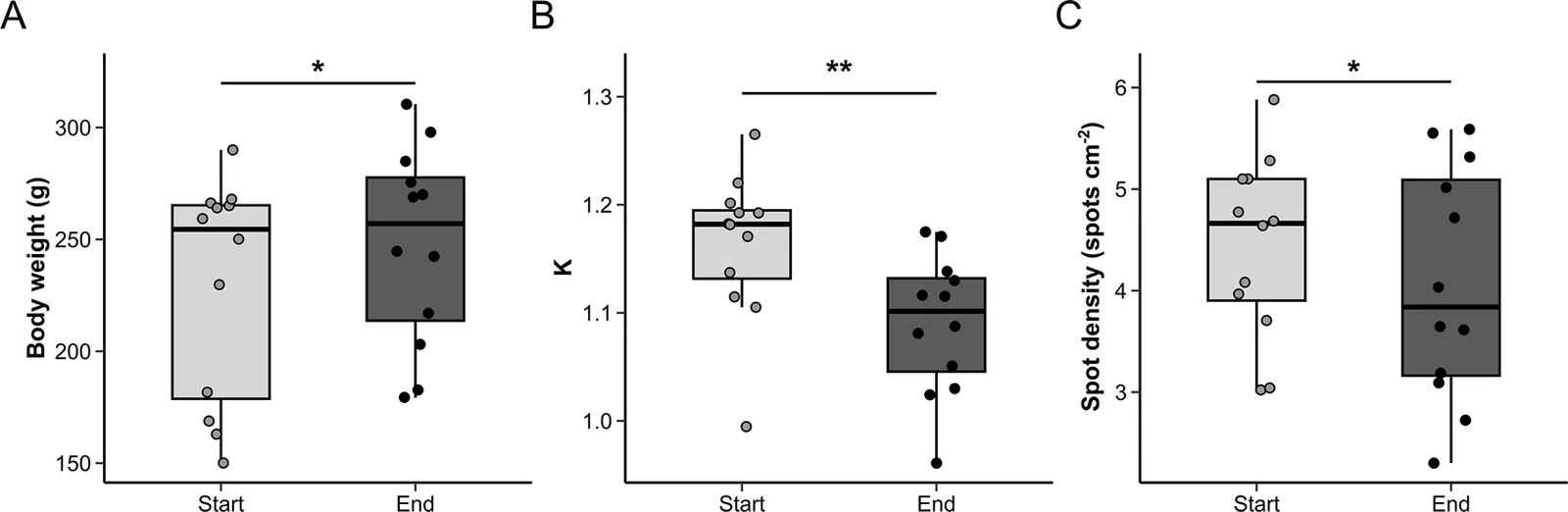
Biometric changes from start to end of the experiment. (A) Body weight (g; Wilcoxon signed-rank test), (B) condition factor (*K*; paired *t*-test), and (C) skin spot density (spots cm^−2^; paired *t*-test). Individual values are shown as points (*n* = 12). Boxes represent interquartile ranges, horizontal lines indicate medians, and whiskers denote data range. Asterisks indicate statistical significance (*p < 0.05, **p < 0.01).

### 3.2. Post-transfer pigmentation change reflects performance

Skin spot density generally declined over the observation period. However, the magnitude of this decline varied substantially among individuals and was associated with performance in the new environment (figure 4). Relative change in spot density was positively associated with growth (% change in body weight; *R*^2^ = 0.71, *b* = 0.96, 95% CI [0.52, 1.39], *p* < 0.001; figure 4A), cumulative feed intake (% of initial bodyweight; *R*^2^ = 0.76, *b* = 1.82, 95% CI [1.09, 2.54], *p* < 0.001; figure 4B) and change in condition factor (% of initial *K*; *R*^2^ = 0.55, *b* = 1.69, 95% CI [0.61, 2.77], *p* = 0.006; figure 4C). Individuals with lower performance exhibited pronounced loss of skin spot density, whereas individuals with higher performance showed reduced loss of spots. Positive changes in spot density were restricted to a few individuals with the highest growth rates. Together, these bivariate relationships show that individuals with higher post-transfer performance generally retained more visible skin spots, with the strongest patterns observed for feed intake and growth.

**Figure 4.**
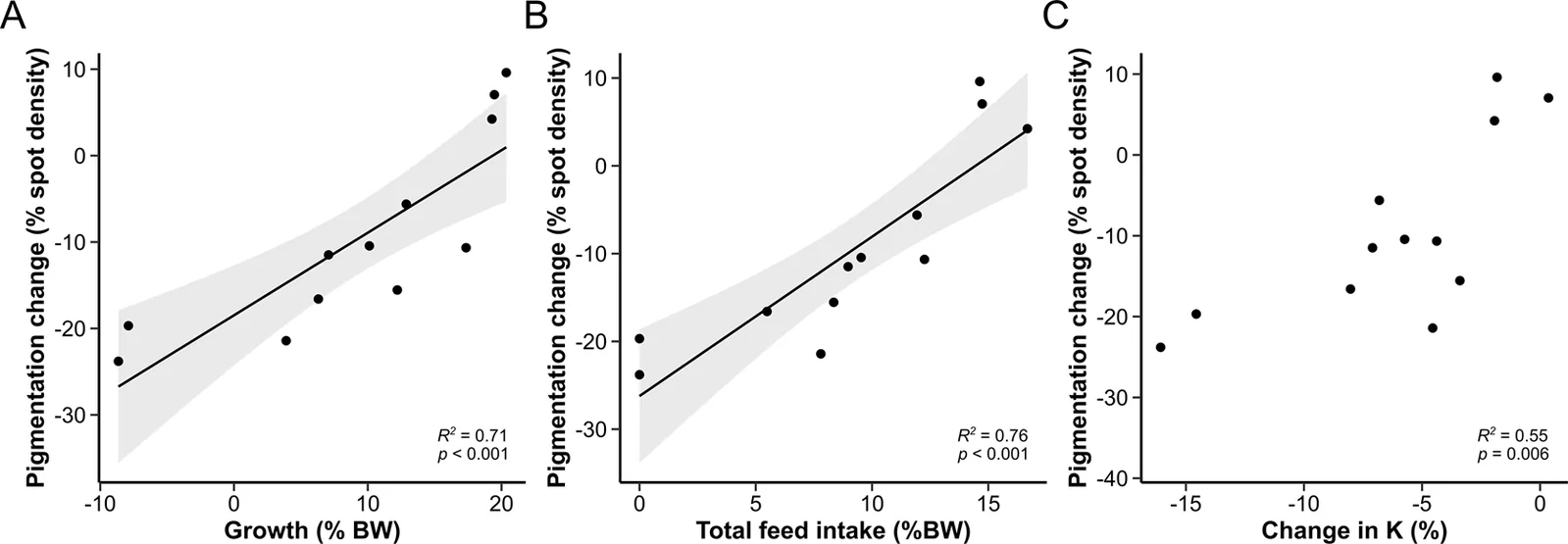
Bivariate relationships between individual performance metrics and relative changes in melanin-based skin pigmentation. Relationships are shown between (A) growth (% change in body weight), (B) cumulative feed intake (% of initial body weight), and (C) change in condition factor (%) and relative change in skin spot density (%). Lines in (A) and (B) indicate linear fit; shaded areas represent 95% confidence intervals (CIs). The regression line and CI are omitted from panel (C) because change in condition factor did not explain unique variation in pigmentation change after accounting for initial body weight. Points represent individual fish (*n* = 12). Adjusted analyses accounting for initial body weight are presented in table 1.

### 3.3. Baseline pigmentation as a predictor of post-transfer performance

To assess whether the relationship between performance and pigmentation change could be anticipated from baseline phenotype, we next examined whether initial spot density predicted post-transfer performance. Skin spot density (spots cm^−2^) at the start of the observation period was associated with individual performance following transfer to the novel environment (figure 5). Fish with higher baseline spot density exhibited higher growth (*R*^2^ = 0.43, *b* = 7.29, 95% CI [1.41, 13.20], *p* = 0.020; figure 5A), higher cumulative feed intake (*R*^2^ = 0.45, *b* = 4.08, 95% CI [0.93, 7.22], *p* = 0.016; figure 5B) and a smaller decline in condition factor (*R*^2^ = 0.34, *b* = 3.23, 95% CI [0.08, 6.39], *p* = 0.046; figure 5C). By contrast, no significant relationships were found between baseline spot density and initial body weight or initial *K* (*p* = 0.17 and 0.34, respectively). These results indicate that baseline pigmentation was associated with subsequent performance following exposure to environmental novelty.

**Figure 5.**
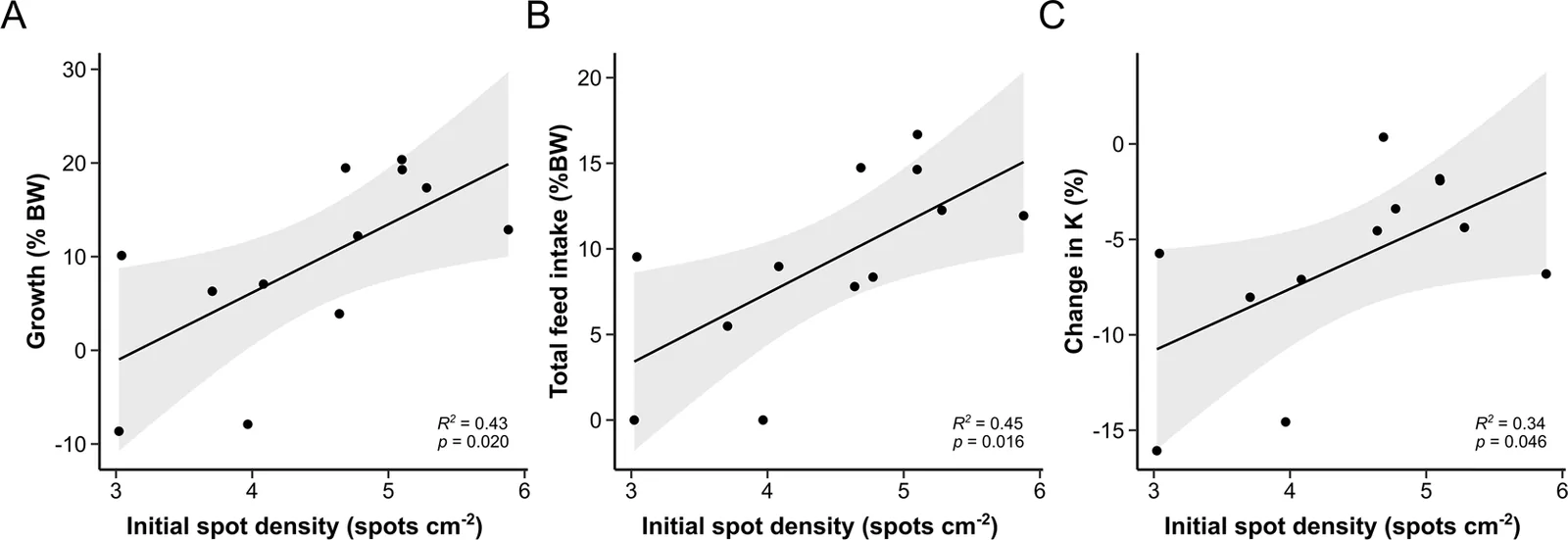
Linear relationships between initial skin spot density and post-transfer performance. Relationships between skin spot density (spots cm^−2^) at the start of the experiment and (A) growth (% change in body weight), (B) cumulative feed intake (% initial body weight), and (C) change in condition factor (%). Solid lines indicate linear fits with 95% confidence intervals (shaded areas). Each point represents individual fish (*n* = 12).

### 3.4. Performance–pigmentation associations accounting for initial body weight

To assess whether associations between performance variables and pigmentation change were independent of initial body weight, models including both initial body weight and individual performance metrics were evaluated (table 1). Growth remained positively associated with relative change in skin spot density when initial body weight was included in the model (*p* = 0.017). Similarly, accumulated feed intake remained positively associated with relative change in skin spot density after accounting for initial body weight (*p* = 0.005). By contrast, change in *K* did not explain unique variation in pigmentation change after accounting for initial body weight (*p* = 0.130), although the overall model, including both initial body weight and change in *K*, remained significant (*p* = 0.016). Initial body weight did not explain unique variation in pigmentation change in any of the three models after accounting for the corresponding performance metric.

**Table 1.** Multiple linear regression models testing associations between performance metrics and pigmentation change while controlling for initial body weight. (Note: *b* denotes the unstandardized regression coefficient, s.e. the standard error of *b*, and LL and UL the lower and upper limits of the 95% confidence interval (CI). Overall model fit (adjusted *R*^2^) is reported in the row corresponding to the dependent variable, with individual predictor estimates listed below. Statistical significance is indicated as *p < 0.05, **p < 0.01 and ***p < 0.001.)

			95% CI for *b*				
dependent variable	predictor	*b* ± s.e.	LL	UL	test statistic	adjusted *R*^2^	*p*
spot density change	start weight + growth				*F*_2,9_*=* 12.32	0.67	0.003**
	start weight	−0.05 ± 0.05	−0.17	0.07	*t*_9_ = −0.94		0.373
	growth	0.78 ± 0.27	0.17	1.39	*t*_9_ = 2.91		0.017*
spot density change	start weight + feed intake				*F*_2,9_*=* 17.47	0.75	<0.001***
	start weight	−0.06 ± 0.04	−0.16	0.04	*t*_9_ = −1.27		0.235
	feed intake	1.50 ± 0.40	0.59	2.41	*t*_9_ = 3.72		0.005**
spot density change	start weight + change in *K*				*F*_2,9_ = 6.85	0.52	0.016*
	start weight	−0.08 ± 0.07	−0.23	0.08	*t*_9_ = −1.11		0.297
	change in *K*	1.15 ± 0.69	−0.41	2.70	*t*_9_ = 1.67		0.130

### 3.5. Sex differences

No differences were detected between males and females in relative change in spot density, growth, cumulative feed intake or change in condition factor (all *p* > 0.05).

## 4. Discussion

### 4.1. Summary of main findings

Juvenile Atlantic salmon exhibited substantial individual variation in changes in melanin-based spot density following transfer to a novel environment. Although most fish showed a reduction in visible spots, the magnitude of this change covaried consistently with performance. Individuals with higher feed intake, greater growth and smaller declines in condition factor exhibited reduced loss of spots and in some cases increased spot density. Baseline spot density further predicted post-transfer performance.

### 4.2. Condition-dependent modulation of melanin-based skin pigmentation

The observed relationships between performance metrics and within-individual changes in visible spot density support the view that melanin-based skin pigmentation can show condition-associated variation, despite often being described as relatively stable and strongly influenced by genetic factors. Individuals with higher growth, greater feed intake and smaller declines in condition factor exhibited reduced loss, or even gains, in visible spots, whereas lower-performing individuals showed pronounced reductions. The positive association between growth and spot density change argues against passive dilution of fixed spots across an expanding body surface and is more consistent with regulated changes in visible pigmentation or spot detectability. This convergence across multiple performance measures suggests that pigmentation dynamics reflect individual state and robustness when faced with changes in the environment rather than simple variation in body size alone.

Melanin-based pigmentation is increasingly recognized as part of an integrated physiological system, shaped by pleiotropic links between pigmentation pathways and endocrine, metabolic, and stress-related processes [36,38]. In vertebrates, melanin expression is closely tied to the melanocortin system, which also regulates energy balance, stress responsiveness, immune function and behaviour [39,40,106,107]. In fishes, the melanocortin system is likewise involved in pigmentation and broader physiological regulation [108]. These shared regulatory pathways provide a plausible framework through which variation in feed intake, growth and condition factor could covary with visible spot expression in Atlantic salmon.

Beyond pleiotropic regulation, melanin-based pigmentation may also be influenced by energetic and nutritional constraints [109]. Although melanin can be synthesized de novo, its production and maintenance depend on dietary precursors, including aromatic amino acids, metals and fatty acids [43], and may therefore be subject to allocation trade-offs with other energetically demanding processes such as growth, immune function and tissue maintenance. Under limited intake or elevated energetic demand, investment in pigmentation may be reduced in favour of essential physiological functions, whereas individuals with greater energetic capacity may retain or enhance melanin expression [110,111].

In the present study, all individuals experienced a transient period of feeding suppression following transfer to individual housing but differed markedly in their latency to resume feeding, a central behavioural aspect of varying coping styles [112] and ability to sustain growth. The divergence in pigmentation dynamics, whereby most individuals lost visible spots while only the highest-performing individuals increased spot density, suggests that maintaining or enhancing spot expression may require exceeding a performance- or energetic threshold under environmental challenges. Such dependent responses are consistent with condition-dependent trait expression, where only individuals in sufficiently good condition can invest in secondary phenotypic traits.

Condition-dependent expression of melanin-based pigmentation is well documented in vertebrates, including associations with individual state, physical performance, food availability and dietary limitations of melanogenic precursors [40,41,53,63–65,71,75,113]. In fishes, including salmonids, melanin-based skin pigmentation has been linked to stress responsiveness, coping style, infection resistance, growth-related traits and environmental conditions, including background colour and rearing environment [80,88,89,95,96]. The present findings extend this work by showing that visible dorsal skin spot density changed within individuals over time in juvenile Atlantic salmon, and that the magnitude of this change covaried with feed intake, growth and condition factor following transfer to individual housing. While the study does not directly test underlying mechanisms or signalling function, the observed relationships are consistent with dynamic, condition-associated regulation of visible spot expression.

A central question is whether the observed pigmentation dynamics primarily reflect intrinsic variation in pigment regulation or emerge secondarily from differences in nutritional state. One possibility is that underlying physiological differences in stress responsiveness or endocrine regulation influence both behaviour and pigmentation, such that individuals which resume feeding more rapidly also maintain or enhance melanin expression. Alternatively, behavioural responses to environmental novelty, particularly the trade-off between feeding and risk avoidance, may drive differences in nutritional intake, which, in turn, constrain or facilitate pigment expression. Under this scenario, pigmentation dynamics would reflect downstream consequences of energy balance rather than direct physiological regulation. The present data do not allow these alternatives to be disentangled, but both pathways are consistent with the observed coupling between feeding performance and changes in visible spot density.

### 4.3. Performance, coping style and possible underlying mechanisms

Individual variation in feeding recovery following transfer to individual housing provides a behavioural context for the observed performance–pigmentation relationships. Such variation is widely interpreted as reflecting differences in coping ability or stress responsiveness in fishes [98,114] and, in salmonids, is linked to feeding motivation, stress sensitivity and post-disturbance growth [112,115]. Consistent with this framework, baseline pigmentation predicted subsequent performance. Given established links between melanin-based spottiness and stress responsiveness in salmonids [88], baseline spot density may index underlying differences in stress physiology or behavioural strategy that influence responses to environmental disturbance. Although most individuals exhibited reduced spot density following transfer, the magnitude of this change varied with performance, arguing against a generalized stress-induced depigmentation response. Instead, individuals with faster feeding recovery and higher growth showed reduced loss of spots or even gains in visible spot density, suggesting that trade-offs between growth and risk avoidance may be resolved differently among individuals under environmental challenge [116–118].

Initial body weight was considered as a potential factor influencing pigmentation dynamics because body size may covary with feeding and growth responses following transfer. However, when initial body weight was modelled together with the performance metrics, it did not explain unique variation in pigmentation change. Growth and feed intake remained associated with pigmentation change after accounting for initial body weight, whereas change in *K* did not. One possible explanation is compensatory growth, whereby individuals with constrained prior growth exhibit accelerated growth when conditions improve [119]. Even in the absence of explicit feed restriction, growth priorities may differ across size classes, particularly during juvenile stages when rapid growth can influence competitive ability, risk sensitivity and survival [120,121]. While these mechanisms cannot be distinguished here, the adjusted models support the interpretation that pigmentation dynamics tracked performance rather than body size *per se*. Consistent with previous work linking higher spottiness to lower stress responsiveness in salmonids [88], both baseline spottiness and its dynamic modulation covary with post-transfer performance.

### 4.4. Visual contrast, background adaptation and signal detectability

An important consideration when interpreting changes in spot density is that our analysis quantified the number of visually detectable melanin-based spots within a standardized ROI, rather than the melanin content or melanophore density. Following transfer from white rearing tanks to grey-background aquaria, fish exhibited gradual darkening of overall skin coloration consistent with background adaptation responses described in teleost fishes [80]. Consequently, a reduction in counted spot density may partly reflect decreased visual contrast between discrete spots and surrounding skin, rather than complete loss of localized melanophores.

However, pigmentation dynamics in salmonids are not governed solely by environmental background. Social context and dominance status can strongly modulate pigmentation, with subordinate individuals often exhibiting overall darkening that can override background matching [122–125]. Such changes are mediated by endocrine mechanisms linked to stress responsiveness and the melanocortin system [126], demonstrating that pigmentation is actively regulated and responsive to internal state, rather than passively determined by environmental cues alone. Although fish in the present study were housed individually, previous work indicates that physiological state can modulate or override background adaptation responses [125], suggesting that the variation in spot detectability probably reflects differences in internal regulatory capacity, including the ability to maintain contrast between spot markings and surrounding skin during background adaptation.

From a signalling perspective, trait expression is functionally meaningful only insofar as it can be perceived by receivers [127]. Reduced contrast diminishes detectability and thus the potential informational value of melanin-based spot patterns, regardless of underlying biochemical pigment retention. Although the specific signalling role of melanin-based spots in salmonids remains unresolved, visual displays are central to social interactions and hierarchy [125,128], and changes in contrast may therefore influence how pigmentation patterns are perceived during social encounters.

The observed pattern, whereby low-performing individuals exhibited pronounced reductions in visible spots, while high-performing individuals retained or enhanced spot visibility, further suggests that contrast maintenance may not be purely automatic. Morphological colour change involves regulated pigment redistribution within melanophores and is influenced by endocrine and metabolic state [80,129]. Individuals under energetic constraints or heightened stress may prioritize core homoeostatic processes over the maintenance of high contrast visual patterns. This is consistent with energetic allocation trade-offs, whereby limited resources are prioritized towards essential physiological functions at the expense of secondary phenotypic expression. Although this mechanism was not directly tested in the present study, reduced spot detectability may reflect differential regulatory capacity rather than unavoidable pigment loss.

The present data do not establish whether salmon assess spot patterns visually or whether spot contrast influences social interactions. Resolving the perceptual, physiological and signalling components will require studies combining behavioural assays, visual perception analyses and direct pigment quantification.

### 4.5. Limitations

Several aspects of the present study warrant cautious interpretation. Although effect sizes were substantial and consistent across performance metrics, replication in larger and more heterogeneous populations is required to confirm the robustness of these relationships.

The analytical approach relied primarily on simple and two-predictor linear regression models rather than a comprehensive multivariate framework. This strategy was necessary to avoid overparameterization given the limited sample size and to address specific biological hypotheses separately. However, performance metrics, such as feed intake, growth and change in condition factor, are physiologically interrelated and not statistically independent. Accordingly, the observed associations are best interpreted as reflecting coordinated performance phenotypes (i.e. indicators of individual condition) rather than strictly independent predictors. While patterns were consistent with shared associations between body size, performance and pigmentation, formal mediation analyses were not conducted, and causal pathways cannot be definitively established.

Pigmentation was quantified as the number of visually detectable spots within a standardized ROI, rather than through direct measurement of melanin content or melanophore density. Because fish underwent gradual background-induced darkening following transfer, reductions in spot counts may partly reflect changes in visual contrast rather than absolute pigment loss. In addition, images were obtained under non-standardized lighting conditions, which may influence absolute contrast. However, analyses were based on within-individual comparisons and standardized contrast enhancement (CLAHE), reducing sensitivity to variation in illumination across samples. Together, these factors primarily affect interpretation of proximate mechanisms rather than the observed association between pigmentation dynamics and performance.

No significant sex differences were detected in pigmentation dynamics or performance, although the juvenile stage of the fish and lack of reproductive investment probably limited the potential for sex-specific divergence. Furthermore, the study was restricted to individually housed individuals, precluding the examination of social effects on pigmentation.

### 4.6. Implications and future directions

Future work should manipulate social context while controlling for feeding and growth to disentangle the relative contributions of social interactions and individual performance to pigmentation dynamics. These findings highlight substantial short-term plasticity in spot density within a commercial stock, emphasizing the need for integrative approaches that combine physiological, behavioural and environmental perspectives.

In particular, longitudinal endocrine sampling coupled with standardized stress tests would help resolve the temporal relationship between neuroendocrine responses and pigmentation dynamics. Experimental manipulation of background coloration under controlled feeding regimes would further clarify the relative contributions of environmental matching and internal regulation to changes in visible spot contrast. Incorporating behavioural assays of coping style, risk-taking and social interaction will be essential to determine the extent to which melanin-based spot patterns both reflect and influence behavioural phenotypes.

Advances in automated analysis and computer vision offer additional opportunities to quantify pigmentation dynamics objectively and at scale. High-resolution imaging combined with automated spot detection and contrast analysis could enable longitudinal monitoring of pigmentation changes in both experimental and applied settings.

## 5. Conclusions

Density of visible melanin-based skin spots in juvenile Atlantic salmon changed following transfer to a novel environment, with both the magnitude and direction of change varying among individuals. This variation was associated with differences in feed intake, growth and condition factor. Baseline spot density also predicted post-transfer performance, linking initial pigmentation state to subsequent variation in these traits. Together, these findings support the interpretation that melanin-based skin spots in Atlantic salmon can show condition-associated variation, with both baseline expression and within-individual changes reflecting variation in individual performance during acclimation. These results indicate that dynamic changes in externally visible pigmentation can track individual responses to environmental challenge.

## Data Availability

The datasets, image files and R code supporting this study are available in the DataverseNO at [133].
